# Potential Mutations Associated With Occult Hepatitis B Virus Status

**DOI:** 10.5812/hepatmon.15275

**Published:** 2014-05-03

**Authors:** Sima Besharat, Aezam Katoonizadeh, Abdolvahab Moradi

**Affiliations:** 1 Liver and Pancreatobiliary Diseases Research Center, Digestive Disease Research Institute, Tehran University of Medical Sciences, Tehran, IR Iran; 2 Golestan Research Center of Gastroentrology and Hepatology, Golestan University of Medical Sciences, Gorgan, IR Iran

**Keywords:** Hepatitis B, Mutation, Virus Diseases

## Abstract

**Context:**

Occult hepatitis B virus (HBV) status (OHBS) is simply defined as the presence of HBV DNA in the liver (with or without detectable HBV DNA in the serum), in the absence of serum HBV surface antigen (HBsAg). Importance of OHBS is mostly clinical, related to its possible role in spreading through blood transfusion and liver transplantation; causing classic forms of HBV. Mechanisms underlying this entity are poorly defined. Several possibilities have been suggested, with major classification into two groups: defective host immune response and viral replication activity through mutations of HBV DNA sequence. Mutations are extensively investigated in all four overlapping open reading frames (ORFs) of HBV genome, to define their possible role in the pathogenesis of OHBS. Some of these mutations like S-escape mutants could not be detected by the routine available assays, making them difficult to diagnosis. Therefore, trying to detect this covert condition could be more helpful for defining better preventive and therapeutic strategies.

**Evidence Acquisition:**

In the present study we provided an in-depth review of the most important new data available on different mutations in HBV genome of patients with OHBS, which may play a role in the pathogenesis of OHBS. The data were collected through reviewing the full-text articles, identified by the PubMed search, using the following keywords and their different combinations: occult hepatitis B, HBV genome, "a" determinant, HBV open reading frames, S mutations, X mutations, P mutations and C mutations.

**Results:**

Variants within the major hydrophilic region (MHR) of the HBsAg, deletions in the pre-S1region, codon stop in the S open reading frames (ORF), sporadic non common mutations, some mutations affecting the posttranslational production of HBV proteins in the S ORF like deletion mutations, mutations in start codon and nucleotide changes in the X ORF, deletion and point mutations in P ORF and sometimes, nucleotide substitution in the C ORF are among the assumed mutations detected to have a role in OHBS appearance.

**Conclusions:**

Studies mostly lacked a control group and the whole-length HBV sequencing was scant with conflicting results, suggesting that OHBS is often a result of multiple mechanisms. Additional studies on full-length HBV genomes from occult and non-occult HBV cases may shed more light on the interplay between different mechanisms involved in the pathogenesis of OHBS.

## 1. Context

Hepatitis B virus (HBV) infection is a global health problem, affecting more than 2 billion people worldwide, of whom approximately 350 million suffer from HBV-induced chronic liver diseases (1, 2). Depending on the interactions between the host and the virus, the natural course of HBV infection can be highly heterogeneous (3). Chronic HBV infection is diagnosed by detection of serum hepatitis B surface antigen (HBsAg), however, sometimes HBV infection can be presented in the absence of serum HBsAg, which is known as occult HBV status (OHBS). Accordingly, OHBS is characterized by the presence of HBV DNA in the liver, in the absence of serum HBsAg, with or without detectable HBV DNA in the serum. On the basis of HBV antibody proﬁle, OHBS may be distinguished as: seropositive-OHBS (anti-HBc and/or anti-HBs positive) and Seronegative-OHBS (anti-HBc and anti-HBs negative) (4).The clinical relevance of OHBS has not been investigated extensively; however, several studies have suggested a potential association between OHBS and increased risk of cirrhosis and hepatocellular carcinoma (HCC). In addition, it can be transmitted throughliver transplantation or blood transfusion (2, 5, 6).

During HBV replication, HBV DNA transcription occurs via a reverse transcriptase enzyme, which performs both priming and elongation activities. Viral reverse transcriptase lack of proof-reading ability, leads to the emergence of different mutations (5, 7). The seronegativity in patients with OHBS may be due to mutations happening through different mechanisms, including changing the immunoreactivity of different viral proteins and the serum level of HBsAg (7).

### 1.1. Virus Description

**Figure 1.**
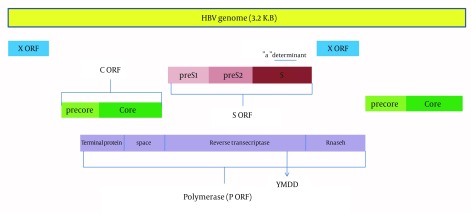
A schematic Figure of HBV Genome and the Four Open Reading Frames

HBV is a small enveloped virus, containing partially double-stranded DNA. The HBV DNA genes are transcribed in four different open reading frames (ORFs). There are four partially overlapping ORFs encoding seven different HBV proteins. The largest ORF is the POL ORF, which encodes polymerase proteins. The S ORF comprises the pre-S1, pre-S2 and S regions and codes for large, middle and small sized inter-membrane surface proteins. The C ORF consists of pre core and core regions and codes the capsid (core) and the hepatitis B e antigen (HBeAg) proteins. HBeAg seems to have a role in the regulation of the immune response. The last ORF is X, which encodes the X protein. The transcription regulation activity of this protein has been suggested by some investigators (Figure 1) (5, 8-14).

Mechanisms underlying OHBS are poorly understood. Several possibilities have been suggested including:

- interference of HBV replication by other viruses (like HCV in case of HCV coinfection),
- integration of HBV-DNA into host cell chromosomes,
- formation of circulating HBV-containing immune complexes, which are not detected by routine HBsAg determining tests,
- altered host immune responses leading to the maintenance of HBV infection in a latent state until transmission to another individual occurs (mostly in case of immunosuppressive therapy),
- different mutations in HBV DNA sequence which is the main focus of the current review and will be discussed in detail (Table 1) (2, 6, 15).

**Table 1.** Studies Investigating Mutations Associated With Occult Hepatitis B Virus Status

Affected Region	First Author	Year	Journal Name	Sample	Main Mutations Found to be Responsible for OHBS
**S region**					
	Hou et al. (16)	2001	Hepatology	OHBS patients	G145R mutation, some positions inside and outside the “a” determinant
	Ma et al. (17)	2012	J Med Virol	OHBS patients	G145R mutation, escape mutations mostly in the “a” determinant
	Liu et al. (18)	2010	Virol J	OHBS in blood donors	substitutions in the regions from aa 117 to 121 and aa 144 to 147, located in the MHR and mutants with single-point or multi-point G145R mutations
	Yuan et al. (19)	2010	JHepatol	OHBS in blood donors	substitutions in the regions from aa 117 to 121 and aa 144 to 147 located in the MHR and mutants with single-point or multi-point G145R mutations
	Panigrahi et al. (20)	2010	Virol J	HBsAg negative blood donors	Single or multiple aa substitutions. Stop codon in a case of HBV (genotype D) with a single nucleotide substitution T to A at nucleotide 207 of the HBsAg and in another case
	Huang et al. (21)	2012	JHepatol	OHBS, HBSAg+ and HBSAg- patients	MHR mutations
	Sengupta et al. (22)	2007	J Med Virol	OHBS patients and a group of HBV carriers	mutations in Pre-S1 & Pre-S2/S promoters/Pre-S1 coding regions
	Chen et al. (23)	2011	Virus Res	OHBS serum samples	Deletions covered the pre-S1 start codon and the B-cell antigenic epitope in the pre-S1 protein (aa19–26). Deletion (nt. 3145-52) covering nearly the entire pre-S2 region
	Pollicino et al. (24)	2007	Hepatology	Liver tissue	Occult HBV populations showed a large intra-individual genetic heterogeneity, which was comparable to that observed in HBsAg-positive subjects.(Host factors), detection of YMDD mutated strains in 38% of occult HBV cases
	Chaudhuri et al. (25)	2004	Gastroenterology	OHBS serum samples	Pre-S2/S region deletion
	Bruni et al. (12)	2011	Virol J	Bioinformatic approach	Higher frequency of stop codons in the S ORF with no significant different. Variations in seven nucleotide positions. The P, the L, the M and S and the core proteins, (but not the X), were the targets of the observed significant variations.
	Ito el al. (26)	2010	J Virol	OHBS serum samples	Some sporadic non common mutations are shown to be relevant to OHBS; for example I110M, G119E, and R169P mutations
	Sheldon and Soriano (27)	2008	J AntimicrobChemother	OHBS serum samples	Premature stop codons in the S gene
	Hass et al. (28)	2005	Hepatology	OHBS serum samples	No expression of pre-S2/S mRNA due to a single G-A mutation within the S gene (G458A mutation)
**X region**					
	Fukuda et al. (29)	1996	Microbiol and Immunol	OHBS serum samples	An identical 8-nucleotide deletion mutation at the distal part of the X region affected the core promoter and the enhancer II sequence
	Fujise et al. (30)	2011	World J Hepatol	OHBS serum samples	Start codon in the X region, mutation of the ATG (Methionine) start codon to GTG (Valine)
	Pollicino et al. (24)	2007	Hepatology	OHBS liver tissue	A1762T/G1764A double mutation and in addition to C1766T (triple mutation), point mutations in BCP
**P region**					
	Chen et al. (31)	2012	Virus Res	OHBS serum samples	Deletion located between nt. 2067 and 2349, covered the start codon of the P region
	Fang et al. (32)	2004	World J Gastroenterol	OHBS serum samples	Forty point mutations
**C region**					
	Chen et al. (31)	2012	Virus Res	OHBS serum samples	Two deletions (nt. 2001–2050 and nt. 2152–2222)
	Garcia-Montalvo et al. (33)	2011	Ann Hepatology	OHBS serum samples	Aminoacid substitution in the core region
	Chaudhuri et al. (25)	2004	Gastroenterology	OHBS serum samples	Stop signals in precore and core regions
	Pollicino et al. (24)	2007	Hepatology	OHBS serum samples	G1896A and missense mutation in the core region

## 2. Evidence Acquisition

In the present study we provided an in-depth review of the most important new data available on different mutations in HBV genome of patients with OHBS, which may play a role in the pathogenesis of OHBS. The data were collected through the review of the full-text articles identified by PubMed search, using the following keywords 2and their different combinations: occult hepatitis B, HBV genome, "a" determinant, HBV open reading frames, S mutations, X mutations, P mutations and C mutations.

## 3. Results

### 3.1. Mutations in the S Region of HBV and Occult HBV Status

The S region of ORFs consists of three AUG codons coding the expression of three proteins: large (L), middle (M) and small (S). Pre-S1 domain is unique for L protein. Pre-S2 domain is the shared sequence with the M protein and the S domain is seen in all three proteins. The L and S proteins are essential for virion formation and the M could enhance the virion secretion efficiency (19, 34, 35). The S and M proteins are detected as HBsAg. HBsAg is a peptide with 226 amino acids (aa) with a single major antigenic determinant called the "a" determinant, located in the-amino acid positions between 100 and 160. The dominant epitopes of HBsAg, which are the targets of neutralizing B cell responses, are located in the "a" determinant (aa 124–147) within the MHR. Mutations inducing a conformational change within the "a" determinant cause making a protein with significant changes in the antigenic epitope. These changes lead to the production of the undetectable HBsAg (21).

The most common and problematic MHR mutation, G145R, is an increasing event due to the global implication of vaccination programs and the pressure of antiviral therapy (21). Variants within the MHR of HBsAg were the point of interest in Hou et al. study in China. In 46 cases with OHBS, there were32 amino acid substitutions found between positions 100-160 of the MHR. In addition to the G145R, 11 positions inside and five positions outside the "a" determinant were involved. Combined mutations were also detected in some patients. Another two patients had insertion mutations immediately before the "a" determinant (16).

Ma et al. in a study conducted China, 2012, found other eight escape mutations associated with OHBS, in addition to the G145 R, located mainly at positions 120, 126, 129, 130, 133, 134, 137, 140, 143 and 144 with a genotypic heterogeneity (17). In the aforementioned study, a comparison was performed between OHBS patients and a group of HBV carriers, which could be considered as the strength of this work. Substitutions in the regions from aa 117 to 121 and aa 144 to 147 located in the MHR of the S gene and mutants with single-point or multi-point G145R mutations were also reported in the studies from China (18, 19). One of these studies was a phylogenetic one on blood donors and no comparisons with non-OHBS patients was performed in it (19). Other single or multiple aa substitutions have been reported to be responsible in OHBS (20). Panigrahi et al. compared the 64-160 aaof60 HBsAg (-) samples, with the reference sequences of each genotype, in their study on 729 HBsAg negative donor samples in India. They found single or multiple aa substitutions in 95% of the OHBS cases. T125M was the most common (93.3%) aa substitution found in the MHR, mostly in subgenotype D3. Substitutions were also found at codon A128V, G71 D, L95 S, M103I, P111L, S113A, S114P, S117G, T115I, T116P, T118R, and T127A (20).

In an interesting recent study Huang et al. compared the characteristics of 61 patients with OHBS to 153 HBsAg (+) carriers with low titers of serum HBsAg (HBsAg-L group) and 54 samples with high serum HBsAg (HBsAg-H group). MHR mutations were seen significantly more frequently in OHBS cases (55.7%) compared to the HBsAg-L (34.0%) or the HBsAg-H groups (17.1%). Thirteen representative MHR mutations were observed in patients with OHBS. Four out of the 13 mutations strongly decreased the analytical sensitivity of seven commercial HBsAg immunoassays and 10significantly impaired virion and/or S protein secretion in both HuH7 cells and mice (36). This was a significant study regarding the comparisons.

Besides, several investigations have described mutations clustering in the aforementioned key immunodominant regions of the HBsAg, which are able of decreasing the immune recognition of the virus, structural alteration and various mutations in genomic regulatory regions, leading to a strong reduction of HBsAg expression (4, 5, 7, 22, 24). In a functional survey by Sengupta et al. in India, the production, secretion and localization of surface proteins of HBV were studied in HepG2 cells, transfected with the wild-type and mutant pre-S1 and pre-S2/S promoters of HBV molecular clones 313.1. Their results indicated that transfected cells had reduced HBV surface protein secretion and showed cytoplasmic aggregation of HBV surface proteins. It could be concluded that OHBS may be caused due to mutations inpre-S1 and pre-S2/S promoters/pre-S1 coding region, which leads to reduced secretion of HBsAg, aggregation of HBsAg in the endoplasmic reticulum and HBsAg seronegativity (22). This was one of the few studies performed on HBV molecular clones and transfected hepatic cells, which made them capable of investigating the possible mechanisms causing OHBS, more closely.

Deletions in the pre-S1region and the resulted impaired viral packaging, has also been reported as another mechanism for OHBS. In Chen et al. (23) study, two kinds of deletions were seen covering the pre-S1 start codon and B-cell antigenic epitope in the pre-S1 protein (aa19-26), leading to a decrease in HBsAg and HBV virus particles in the serum. In another subject, a deletion was observed (nt. 3145-52), covering nearly the entire pre-S2 region. In one case, a deletion in preS2-promoter (nt. 3145-52) was identified, covering almost the whole pre-S2 region. It has been known that deletions overlapping this region could decrease the expression of the M protein, which reduces virion secretion. In this study cloning and sequencing the full-length genome of HBV wasonly done on nine healthy young Chinese patients with OHBS, who received neonatal vaccination. Although it was a powerful study due to the full-length genome sequencing, there was not any control group. Pollicino et al. investigated the lack of HBsAg production (or detection) and the inhibition of viral replication as major aspects of OHBS in their study. They studied frozen liver specimens of 17 HBV patients (13 OHBS and four HBsAg (+) patients as a control group). Cloning and sequencing of the pre S-S genomic region was detected in only one case with small in-frame deletion and two more cases, out of 13 patients with OHBS, showed point mutation in preS2 start codon. No important mutation was found in the preS1 region of HBV clones from 16 patients. Large intra-individual genetic heterogeneity was observed in OHBS cases, comparable to the HBsAg (+) subjects. Therefore, the authors concluded that the viral genomic variability does not appear to play a fundamental role in inducing the OHBS and host immune system but probably epigenetic mechanisms can play critical roles (24).

In another complete genome assessment conducted in New Delhi (2004), the major observations were: frequent quasi species variation, deletion in pre-S2/S region affecting the surface promoters (nt. 3025-54) and pre-S protein and truncated precore and core regions, related to the stop signal (25). In these two studies, a whole genome sequencing was done in patients with OHBS, although the first one (Pollicino study) could be considered more valuable due to having a control group of HBSAg (+) samples and also working on the liver tissues.

It seems that mutations introducing stop codons in the S ORF are among the common frequent mutations, not necessarily resulting in OHBS. As Bruni et al. in a bioinformatics approach, showed that although the frequency of stop codons in the S ORF was higher in OHBS than non-OHBS sequences, the difference was not statistically significant. The authors also found that variations in seven nucleotide positions were significantly associated with OHBS. The P, the L, the M and S and the core proteins, but not the X, were the targets of the observed significant variations (12).

Panigrahi et al. found a stop codon in one sample of HBV/D, with a single T to A nucleotide substitution at nucleotide 207 of the HBsAg (20). Some sporadic non common mutations are shown to be relevant to OHBS ,for example: I110M, G119E and R169P mutations which could impair virion secretion (26).

Drug resistance is one of the most problematic issues, mostly resulting from mutations in the S region. A triple mutational pattern (rtV173L + rtL180M + rtM204V) causing lamivudine resistance has recently been shown to enhance HBV replication. Other lamivudine-associated resistance mutations may cause premature stop codons in the S gene, also resulting in impaired secretion of the HBsAg (31).

Finally, some mutations affect post-translational production of HBV proteins, inducing OHBS in some cases, as shown in Hass et al. case report. Genomes from two patients showed a low replication phenotype at the level of RNA encapsidation or HBV DNA synthesis, not attributable to uncommon mutations in the terminal protein domain of P protein. A single G-A mutation was identified within the S gene (G458A mutation), responsible for this effect. The nuclear run-on transcription showed that the G458Amutation acts at the post-transcriptional level (28).

### 3.2. Mutations in the X Region of HBV and Occult HBV Status

X ORF produces the X protein (HBx) and although the exact function of HBx during HBV replication is still unclear, multiple studies suggest that HBx is necessary for viral replication in vivo and in vitro (30, 37-40). Mutations in the X region can involve the regulatory elements that control replication, like the basal core promoter and the enhancer II. Because the basal core promoter overlaps with the X gene in the concomitant reading frame, the A1762T plus G1764A core promoter mutations also cause changes in the X gene at xK130M and xV131I (30, 37-40).

Deletion mutations in X region are found in OHBS patients. Fukuda et al. in a very early study on X gene mutation in Japan, showed an identical 8-nucleotide deletion mutation at the distal part of the X region in a major group of these patients (85.7%). This mutation affected the core promoter and the enhancer II sequence of HBV DNA and created a translational stop codon which truncated the X protein by 20 amino acids from the C-terminal end. All the HBV DNAs had a precore mutation at the 83rd nucleotide, resulting in disruption of HBeAg synthesis (29). In this study, serum HBV DNA from patients with non-B non-C hepatitis (NBNC) was sequenced and compared to that of the patients with alcoholic liver disease and auto-immune hepatitis. Therefore, these results could be very interesting keeping the studied group in mind.

The start codon in the X region could also be mutated and cause OHBS. In a Japanese study, the ATG (methionine) start codon had mutated to GTG (valine) and resulted in OHBS in one case (30). Fujise sequenced the full genome of HBV in this seronegative case of OHBS, which is worth giving more attention. Nucleotide exchange of A1762T and G1764A is another important mutation, which has been suggested to be responsible in OHBS. This was reported in Pollicino's study on a group of 13 OHBS and four cases of overt HBV (the control group). They reported the double mutation of A1762T and G1764A in 4 OHBS cases and three controls. Triple mutation of these two plus C1766T was only observed in two OHBS cases. Point mutations (from 1 to 4) in BCP were also reported in the mentioned groups (24). The results of Pollicino's study are valuable regarding the comparison they made between OHBS cases and overt patients with HBV from the point of potential mutations assumed to be responsible in OHBS.

### 3.3. Mutations in P Region and Occult HBV Status

One of the regions with mutation susceptibility in HBV ORF is P region, which encodes the polymerase protein (reverse transcriptase) or the POL. The HBV genome is organized in a way that the envelope (S) gene is completely over-lapped by the polymerase gene, so it is logical to assume that changes in virus encoding, associated with antiviral resistance in the polymerase, may have consequent changes on the envelope gene (27), showing a close relationship between mutations in S and P regions of HBV genome. 

This region is also susceptible to deletion mutations and having a key role in HCC progression. In a full-length genome study of HBV DNA in China, 14 out of the 16 clones, constructed from 3 cases of genotype B showed deletions in the P region. These deletions were located between nt. 2067 and 2349, covering the start codon of the P region, which is believed to reduce the enzymatic activity of the wild-type protein and may be accounted for low viral loads in OHBS (31). Forty point mutations in polymerase gene were found, resulting in changes in 11 amino acids in one case of OHBS, in a study by Fang et al. conducted in a high endemic area for HCC, in China in2004 (32). Therefore mutations in this region should draw the attention to the importance of related drug resistance and hepato carcinogenesis.

### 3.4. Mutations in C Region and Occult HBV Status

C ORF of HBV genome encodes core protein and HBeAg (8-10). The core shell of hepatitis B virus is a potent immune stimulator, stimulating a strong neutralizing immune response to foreign epitopes (39, 40). Mutations in this region of HBV genome have not been assumed to be responsible for OHBS, as frequently as other regions spoken above, therefore there are not as many studies done on the subject. In one study in China BCP deletion mutation was investigated in three clones from one case (nt. 1754, nt. 1751, and nt. 1754). The deletions in the BCP region covered more than one TA box. In the C region, deletions were observed in 4 subjects. Among 14 strains with deletions in the C region, 11 had deletions in all parts of both the C and P regions, all in cases with genotype B. In one case, two deletions (nt. 2001–2050 and nt. 2152–2222) covered 22% of the C region (31).

In another study Garcia-Montalvo et al. reported 24 (6.4%) cases with OHB Samong 372 Mexican blood donors. Phylogenetic analysis in this subgroup showed aa substitution in the core region of nine samples, mostly located in immune dominant epitopes. There was no precore stop codon mutants in these patients (33). Truncated precore and core mutations, resulting in stop signals were found in another study in India on patients diagnosed as OHBS, who were not on anti-viral treatment (25). All mentioned studies were observational surveys on a group of patients with OHBS, looking for mutations in the specific region of HBV genome.

On the other hand, Pollicino et al. in a whole genome study on OHBS and overt HBV cases reported G1896A nucleotide mutation, resulting in a stop signal at codon 28, within the precore region, which prevents the HBeAg synthesis in eight out of 13 OHBS cases and in all 4 overt HBsAg (+) cases (control group). Missense mutations within different core antigen immunogenic epitopes were also observed in HBV isolates, in both patients with OHBS and the control group with overt HBV infection (24).

## 4. Conclusions

OHBS is a complex clinical entity documented worldwide. HBV sequences from these individuals demonstrate numerous mutations/deletions and alterations that can result in decreased immune recognition of the virus, impaired HBV packaging and decreased HBsAg expression. Moreover, mutations affecting post-translational protein production and treatment-associated mutations are observed in these patients. However, the aforementioned studies mostly lacked a control group. In addition, whole-length HBV sequencing data, resulting in direct comparison of mutations between the genome sequences of occult and non-occult strains, even though scant, have conflicting results suggesting that OHBS is often a result of multiple mechanisms. Additional studies on full-length HBV genomes from occult and non-occult HBV cases may shed more light on the interplay between different mechanisms involved in the pathogenesis of OHBS. Such insights are of utmost importance to develop new therapeutic strategies.
